# Mathematical Modeling in Systems Biology

**DOI:** 10.3390/e25101380

**Published:** 2023-09-25

**Authors:** Pavel Kraikivski

**Affiliations:** Academy of Integrated Science, Division of Systems Biology, Virginia Polytechnic Institute and State University, Blacksburg, VA 24061, USA; pavelkr@vt.edu

Mathematical modeling is a key tool used in the field of systems biology to determine the mechanisms with which the elements of biological systems interact to produce complex dynamic behavior. It has become increasingly evident that the complex dynamic behavior of biological systems cannot be understood by intuitive reasoning alone. However, valuable insights with regard to the mechanisms governing the dynamic behavior of biological systems can be revealed through computational experiments by simulating these systems with mathematical models. The eleven contributions of this Special Issue demonstrate how computational and mathematical approaches can simultaneously be used to reveal important aspects of various biological problems.

Ute Deichmann presents a comprehensive analysis of the historical background of pattern formation models that are applied to describe morphogenesis and embryological structure development [1]. The analysis tracks the development of physical–chemical and genome-based pattern formation models and subsequently compares Alan Turing’s 1952 reaction–diffusion-based models with more recent models that integrate gene regulatory networks with physical–chemical processes. The article concludes that Turing’s models alone are not able to rigorously explain pattern generation in morphogenesis, but that mathematical models combining physical–chemical principles with gene regulatory networks, which govern embryological development, are the most successful in explaining pattern formation in organisms.

An information-based approach to quantify geometrical order in biological organizations using varying levels of information is introduced in the article by Juan Lopez-Sauceda et al. [2]. The approach employs Shannon entropy to measure the quantity of information in geometrical meshes of biological systems. The authors apply their approach to quantify spatial heterogeneity in thirty-five biological and non-biological geometric aggregates and conclude that the differential entropy of geometrical organizations is an essential source of information in biological systems.

Steven Frank uses reservoir computing techniques to study how a biological system with an internal, randomly connected network receiving environmental inputs evolves to generate predictive responses [3]. The environmental inputs are generated using a mathematical model that exhibits chaotic dynamics. The biological system that interacts with the environment is represented by a random network reservoir that retains the memory of past inputs. The study quantifies the degree of effectiveness and accuracy with which the biological system predicts future input values, using the internal reservoir states as predictors.

The article by Yolocuauhtli Salazar et al. presents a mathematical model of biomass growth, glucose consumption, and ethanol production by *K. marxianus* yeast strains [4]. The model consists of three coupled, nonlinear first-order ordinary differential equations and ten parameters that can be well-constrained by experimental data. The model is successful in explaining the time-course data of alcoholic fermentation in batch culture for 17 different *K. marxianus* strains, and can be used to accurately predict the evolution of both biomass and ethanol in the system.

The review article by Madhumita Srinivasan, Robert Clarke, and Pavel Kraikivski presents mathematical models of different cell death execution mechanisms that have been published over a period of twenty-two years [5]. The authors put forward a hypothesis that cell death can be controlled by a singular, highly integrated cell death decision network that enables cells to choose alternative cell death execution pathways within a single control network of cell death.

Francesco Cordoni’s work presents a macroscopic deterministic approximation of microscopic systems, which is represented by a stochastic model for radiation-induced DNA damage kinetics and repair [6]. The approximation is used to compute the distribution of the number of DNA damages that result in cell death. It concludes that the distribution deviates from the Poisson law due to the clustering of the DNA damage.

The study conducted by Om Prakash et al. is related to DNA-based computing, which relies on error control coding techniques [7]. Coding theory is applied to construct a large set of DNA strings that satisfy certain combinatorial constraints. The authors study reversible DNA codes, as well as those of length *n*, and obtain new DNA codes with improved parameters.

Two articles in this Special Issue focus on the modeling of infection transmission dynamics. The article by Isa Abdullahi Baba et al. presents a fractional-order cholera model that is an extension of the Susceptible–Infected–Recovered epidemic model [8]. The model incorporates the saturated incidence rate to accurately represent the transmission dynamics of the disease. The article by Hosam Alhakami et al. uses a deterministic mathematical model of vector-borne viral plant disease dynamics to train a feed-forward neural network using Levenberg–Marquardt backpropagation algorithm [9]. The neural network is then used to study the implication of fluctuations on natural plant mortality and vector mortality rates.

Mathematical models of biological systems usually describe many interacting components and involve many parameters. Furthermore, it is common that only limited experimental data are available to calibrate the models. Therefore, reliable mathematical models of biological systems can only be developed with rigorous parameter estimation and model validation techniques. Samaneh Gholami and Silvana Ilie propose a parameter estimation method for stochastic discrete models of biochemical networks [10]. The method utilizes finite-difference approximations of the parameter sensitivities and the singular value decomposition of the sensitivity matrix. Several models of biochemical systems are used to demonstrate the advantages of the proposed method.

The article by Christopher Parker, Erik Nelson, and Tongli Zhang presents a computational framework named VeVaPy, which is designed to verify and validate mathematical models comprising many interacting components and parameters [11]. VeVaPy is a publicly available Python library that can help determine which model from the literature is the best for fitting new experimental data. The authors use several hypothalamic–pituitary–adrenal (HPA) axis models from the literature to demonstrate the way in which VeVaPy can help to verify and validate these models against new data: VeVaPy runs the differential evolution parameter optimization algorithm on each model against several novel datasets and ranks the models based on their average cost function value. In their demonstration, two out of five HPA models performed the best in elucidating the novel datasets. Overall, the model validation process is able to operate with significantly less effort when using VeVaPy.

## References

[1] Deichmann U. (2023). Self-Organization and Genomic Causality in Models of Morphogenesis. Entropy.

[2] Lopez-Sauceda J., von Bülow P., Ortega-Laurel C., Perez-Martinez F., Miranda-Perkins K., Carrillo-González J.G. (2022). Entropy as a Geometrical Source of Information in Biological Organizations. Entropy.

[3] Frank S.A. (2023). Precise Traits from Sloppy Components: Perception and the Origin of Phenotypic Response. Entropy.

[4] Salazar Y., Valle P.A., Rodríguez E., Soto-Cruz N.O., Páez-Lerma J.B., Reyes-Sánchez F.J. (2023). Mechanistic Modelling of Biomass Growth, Glucose Consumption and Ethanol Production by Kluyveromyces marxianus in Batch Fermentation. Entropy.

[5] Srinivasan M., Clarke R., Kraikivski P. (2022). Mathematical Models of Death Signaling Networks. Entropy.

[6] Cordoni F.G. (2023). On the Emergence of the Deviation from a Poisson Law in Stochastic Mathematical Models for Radiation-Induced DNA Damage: A System Size Expansion. Entropy.

[7] Prakash O., Singh A., Verma R.K., Solé P., Cheng W. (2023). DNA Code from Cyclic and Skew Cyclic Codes over F_4_[*v*]/<*v*^3^>. Entropy.

[8] Baba I.A., Humphries U.W., Rihan F.A. (2023). A Well-Posed Fractional Order Cholera Model with Saturated Incidence Rate. Entropy.

[9] Alhakami H., Umar M., Sulaiman M., Alhakami W., Baz A. (2022). A Numerical Study of the Dynamics of Vector-Born Viral Plant Disorders Using a Hybrid Artificial Neural Network Approach. Entropy.

[10] Gholami S., Ilie S. (2023). Quantifying Parameter Interdependence in Stochastic Discrete Models of Biochemical Systems. Entropy.

[11] Parker C., Nelson E., Zhang T. (2022). VeVaPy, a Python Platform for Efficient Verification and Validation of Systems Biology Models with Demonstrations Using Hypothalamic-Pituitary-Adrenal Axis Models. Entropy.

